# Recent Advances in Photo-Responsive Microencapsulated Phase-Change Materials

**DOI:** 10.3390/ma18215014

**Published:** 2025-11-03

**Authors:** Chaofeng Yang, Binyang Du

**Affiliations:** State Key Laboratory (SKL) of Biobased Transportation Fuel Technology, Department of Polymer Science & Engineering, Zhejiang University, Hangzhou 310058, China; 22329009@zju.edu.cn

**Keywords:** phase-change materials, microcapsule, photo-responsiveness, multifunctionality

## Abstract

Photo-responsive microencapsulated phase-change materials (MEPCMs) are attracting growing interest for their significant potential in solar energy applications and advanced intelligent thermal management systems, owing to their exceptional capacity for thermal energy storage, efficiency for photothermal conversion, and capability for multifunctional integration. This review provides a systematic summary of the advancements in photo-responsive MEPCMs containing photothermal, photocatalytic, and luminescent materials in the past five years, highlighting their potential in energy conversion, pollutant degradation, and intelligent sensing applications. Moreover, perspectives for future research are provided to enhance the practical application of photo-responsive MEPCMs.

## 1. Introduction

With the continuous growth of global consumption, environmental pollution and energy shortages are becoming increasingly severe, posing higher requirements for energy efficiency [1,2]. Therefore, thermal energy storage (TES) technologies have received widespread attention and emerged as a significant research area [3,4]. Traditional TES technologies are mainly categorized into latent heat storage (LHS) [5,6,7,8,9], thermal chemical heat storage (TCHS) [10,11,12], and sensible heat storage (SHS) [13,14,15]. Compared with SHS (which typically has lower energy density) and TCHS (which depends on chemical reactions), LHS has attracted considerable research attention owing to its favorable safety profile and high energy storage density. Phase-change materials (PCMs) [16,17,18,19], often referred to as LHS materials, exhibit high phase-change enthalpies, enabling them to release and absorb substantial thermal energy with minimal temperature variation during phase transitions.

However, the considerable volumetric variation and leakage tendencies during the phase transition process notably hinder the practical deployment of PCMs, especially the most widely used solid-to-liquid PCMs [20,21]. To solve these challenges, the microencapsulation technique has been widely adopted as an effective approach to stabilize PCMs [22]. This technique not only effectively mitigates the risk of leakage during thermal cycling but also enhances the specific surface area of PCMs, thereby markedly improving their thermal response rate and transfer performance [23]. With the continuous advancements of microencapsulation technologies, increasing efforts have been devoted to incorporating various functional materials into the shell of microencapsulated phase-change materials (MEPCMs), as well as embedding specific nanoparticles in both shell and core regions, aiming to endow MEPCMs with diversified functionalities [24,25,26,27].

In the past few years, the emergence of photo-responsive materials has introduced new opportunities for the advancement of MEPCMs [28]. These materials enhance the utilization efficiency of solar energy and impart additional functional attributes, thereby accelerating the development of MEPCMs toward multifunctionality. This review provides a systematic compilation of the latest research and application status of photo-responsive MEPCMs containing photothermal, photocatalytic, or luminescent materials, with a specific emphasis on their manufacturing techniques, functional mechanisms, recent breakthroughs, and future development directions.

## 2. Microencapsulated Phase-Change Materials (MEPCMs)

MEPCMs feature a core–shell architecture, in which PCMs serve as the encapsulated core, while the protective shell is constructed from inorganic compounds [29,30], polymeric materials [31,32], or metals and their alloys [33]. The microcapsules are prepared via a core–shell strategy. The core, such as paraffin wax, is dispersed in water to form droplets, and a shell—either inorganic (e.g., CaCO_3_), organic (e.g., polymer), or hybrid—is formed around the droplets via in situ polymerization, interfacial polymerization, or sol–gel methods. Functional nanoparticles such as TiO_2_ can be incorporated into the shell to provide additional properties, such as photocatalytic activity. This particulate architecture enables stable and efficient TES. The PCM cores absorb or release substantial thermal energy, thereby facilitating effective energy storage and utilization. PCMs can be divided into two primary categories: organic materials and inorganic materials, depending on their chemical compositions. Organic PCMs primarily include paraffins (saturated hydrocarbons) [34,35], fatty acids [36,37], and their eutectic mixtures [38], as well as polyols. These materials are characterized by chemical inertness, non-toxicity, non-corrosiveness, and a diverse spectrum of phase-transition temperatures, which render them highly appropriate for TES in low- to medium-temperature applications. For instance, *n*-alkanes derived from paraffin waxes exhibit remarkable latent heat capacity, cost-effectiveness, and reliable thermal stability, and they are therefore frequently employed as organic candidates [39]. Apart from paraffin-based materials, non-paraffinic ones such as fatty acids also demonstrate high latent heat, excellent chemical stability, minimal volume variation during phase transitions, and low susceptibility to supercooling. In addition, by blending different types of organic PCMs in specific ratios to form eutectic mixtures, the phase-transition temperature is capable of being flexibly adjusted to meet the demands of specific target applications. However, organic PCMs suffer from inherent drawbacks, including low thermal conductivity, high flammability, and relatively low material density. A lower material density means that less PCM mass is contained within a given volume, resulting in a lower volumetric energy storage capacity. These shortcomings collectively limit their suitability for high-efficiency thermal storage systems. Inorganic PCMs mainly consist of salt hydrates [40] and metals and their alloys [41,42,43,44]. Salt hydrates, composed of water and inorganic salts of crystallization, typically offer remarkable latent heat, moderate thermal conductivity, and low cost, making them one of the most widely utilized categories of inorganic PCMs. Moreover, the relatively remarkable thermal storage density of salt hydrates renders them appropriate for TES applications in the low-to-medium temperature range. However, these materials also exhibit notable drawbacks, including corrosiveness toward metallic components, as well as a tendency to undergo supercooling and phase separation during phase transitions—factors that adversely affect their thermal storage performance and cycling stability [45]. Another class of inorganic PCMs includes metals and their alloys, such as gallium (Ga) and its alloys [46,47] for low-temperature applications, as well as tin (Sn) [48,49], bismuth (Bi) [50], copper (Cu) [51], aluminum (Al) [52,53], etc., for medium- to high-temperature TES. These materials provide excellent thermal conductivity, good reversibility, and high thermal stability during melting and solidification. Therefore, they are regarded as ideal candidates for high energy density and efficient thermal storage. Despite their superior heat transfer performance, metal-based PCMs generally possess lower latent heat per unit mass and involve higher material costs, which limit their feasibility for large-scale TES systems. In general, organic PCMs are more suitable for low-temperature applications where chemical stability, low cost, and non-corrosiveness are required. Inorganic PCMs—particularly salt hydrates and metals and their alloys—are preferred in systems demanding higher thermal conductivity and volumetric energy density. The major differences between organic and inorganic PCMs in terms of their thermophysical properties, advantages, and typical application domains are summarized in Table 1.

MEPCM shell materials can always be fundamentally categorized into inorganic materials and organic materials. Organic shell materials have been broadly adopted owing to their well-established synthesis methods, excellent film-forming properties, and strong encapsulation capability for liquids. Commonly used organic shell materials include melamine-formaldehyde (MF) [54,55,56] and urea-formaldehyde (UF) resins [57,58], polyurethane (PU) [59,60,61], polymethyl methacrylate (PMMA) [31,62], and polystyrene (PS) [63,64,65], etc. Among them, MF resin is the most widely employed in MEPCM fabrication because of its favorable thermal stability, remarkable mechanical strength, and cost-effectiveness. However, organic polymer shells also present certain limitations, including poor thermal resistance, flammability, and relatively low thermal conductivity, which collectively constrain the performance of MEPCMs under high-temperature conditions. Typical inorganic shell materials for MEPCMs include calcium carbonate (CaCO_3_) [29,66,67,68], silicon dioxide (SiO_2_) [69,70,71,72], zinc tungstate (ZnWO_4_) [73], and titanium dioxide (TiO_2_) [74,75,76], etc. These inorganic shell materials possess advantages such as remarkable thermal conductivity, excellent mechanical strength, and non-flammability, which collectively enhance the MEPCMs’ heat transfer efficiency and thermal stability. Such characteristics enhance safety and make them particularly suitable for applications requiring rapid thermal response. Nevertheless, inorganic shell materials also present certain limitations, most notably their poor gas barrier properties and lack of flexibility. These drawbacks may result in the development of microcracks within the shell during the phase transition of the core materials, consequently undermining the structural integrity and long-term cycling stability of MEPCMs. To address the limitations associated with single-shell materials, recent studies have progressively concentrated on creating hybrid shell structures that combine organic polymers with inorganic components [77,78]. Such hybrid shells combine the flexibility of organic materials with the high thermal conductivity and non-flammability of inorganic constituents, thereby enhancing the mechanical robustness and thermal responsiveness of MEPCMs while also improving their heat resistance and structural stability.

Continuous advancements in microencapsulation technology have greatly improved the performance of MEPCMs. Their widespread adoption in energy management, building insulation, textiles, and electronic thermal regulation arises from their outstanding features—high energy storage density, isothermal operation, precise temperature control, and a large specific surface area [63,79,80,81,82,83,84]. As application scenarios continue to diversify, there is a growing demand for functionalized MEPCMs endowed with tailored properties to meet specific performance requirements. To impart additional functionalities to MEPCMs, researchers have explored various strategies, including embedding specific functional nanoparticles into either the core or the shell or utilizing functional materials as the shell structure [26,27,85,86]. These approaches have led to the development of MEPCMs with diverse capabilities such as photo-responsiveness, magnetic responsiveness, antibacterial activity, and self-healing, etc. While retaining excellent thermal regulation performance, these functionalized MEPCMs enable intelligent responses and coordinated control under complex and dynamic conditions, thereby significantly expanding their application potential. Functionalizing MEPCMs by harnessing light as a triggering stimulus or energy input has become a significant area of research, covering various applications such as photothermal conversion [87], photocatalysis [88], and photoluminescence [89]. Among them, photothermal conversion MEPCMs demonstrate the ability to directly harvest light energy and transduce it into storable thermal energy via photothermal conversion mechanisms, which renders them highly suitable for dual-purpose applications involving passive thermal regulation and efficient solar energy harvesting. This functionality is typically achieved by incorporating strong light-absorbing materials, such as conductive polymers, carbon black, metal oxide nanoparticles, or graphene, into the core or shell layers. These functional materials demonstrate strong absorption in the visible-to-near-infrared (vis-NIR) region, enabling efficient conversion of solar energy to thermal energy. Subsequently, the generated thermal energy undergoes rapid convection to the PCMs to trigger their phase transition and facilitate energy capture. In contrast, the realization of photocatalytic functionality in MEPCMs primarily depends on the incorporation of semiconductor materials with photocatalytic activity, such as nanoscale stannic oxide (SnO_2_), cadmium sulfide (CdS), zinc oxide (ZnO), titanium dioxide (TiO_2_), etc. Such photocatalytic MEPCMs not only enable TES but also utilize photogenerated charge carriers (electron–hole pairs) under light irradiation to degrade organic pollutants or drive photochemical reactions, thereby achieving a synergistic integration of TES with pollutant remediation or chemical energy conversion. This strategy effectively integrates solar energy storage with photocatalysis, offering a novel technological pathway for solar-to-chemical energy conversion under the framework of carbon neutrality goals. In addition, photoluminescent MEPCMs are constructed by doping or coating photoluminescent functional materials (like rare earth materials, organic luminophores, and quantum dots, etc.) that emit light upon excitation by specific wavelengths. These photoluminescent MEPCMs not only maintain effective TES capacity but also demonstrate extensive application prospects in areas like architectural aesthetics, smart sensing, and safety indicators.

## 3. Photothermal Conversion MEPCMs

Photothermal materials are capable of efficiently transforming solar energy into thermal energy via their intrinsic physical mechanisms, which typically involve non-radiative transitions, localized surface plasmon resonance (LSPR), or electron–phonon coupling [90]. Based on their composition and structural characteristics, typical photothermal materials can be categorized into carbon-based materials (including carbon black, graphene, and carbon nanotubes, etc.) [91], metal-based materials (including nanoparticles of gold, silver, and platinum, etc.) [92], organic materials (such as indocyanine green, polypyrrole, and polyaniline, etc.) [93,94], and other photothermal agents [95]. Integrating photothermal materials with MEPCMs not only significantly enhances their TES efficiency and thermal conductivity but also imparts excellent photothermal conversion capability.

### 3.1. Carbon-Based Photothermal Materials Integrated with MEPCMs

**Figure 1:**
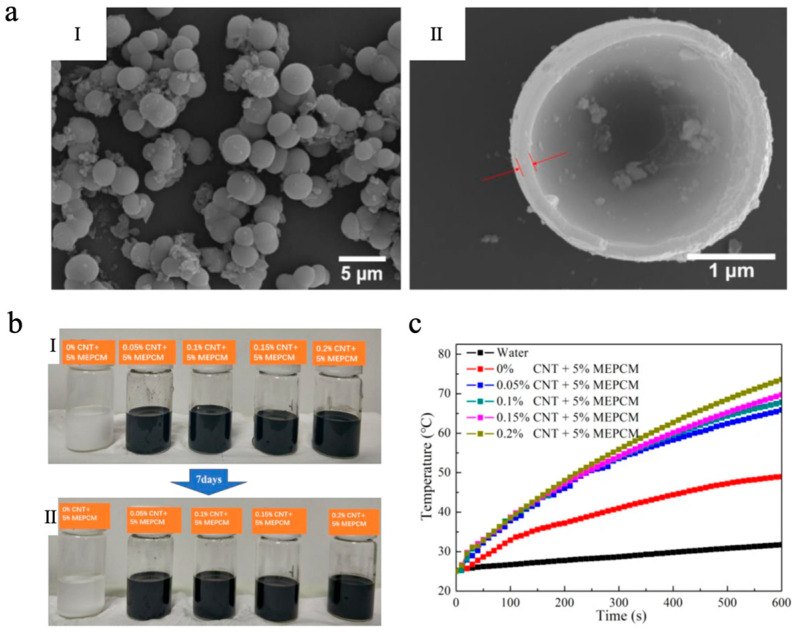
(**a**) SEM images of MEPCMs (I) and the microstructure of MEPCMs (II) [100]. Copyright © 2020, the authors [100] (licensed under: CC-BY 4.0). (**b**) Sedimentation photographs of the suspensions: 0 day (I) and 7 days (II) [100]. Copyright © 2020, the authors [100] (licensed under: CC-BY 4.0). (**c**) Temperature curves of water and mixed suspensions as a function of irradiation time [100]. Copyright © 2020, the authors [100] (licensed under: CC-BY 4.0).

Carbon-based photothermal materials possess broad-spectrum optical absorption characteristics, exhibiting particularly strong absorption in the vis-NIR region [96]. As a result, they have emerged as one of the most widely researched materials for photothermal conversion. Upon absorbing light energy, carbon-based materials undergo electronic excitation from the ground state to excited energy levels, followed by non-radiative relaxation dominated by electron–phonon coupling, which rapidly converts the excitation energy into lattice vibrations, thereby generating thermal energy [97,98,99]. This efficient photothermal conversion pathway enables carbon-based photothermal materials, when integrated with MEPCMs, to significantly enhance the photothermal response rate and solar energy utilization efficiency of the system. Representative carbon-based photothermal materials include carbon black (CB), graphene oxide (GO), reduced graphene oxide (rGO), and carbon nanotubes (CNTs), etc. Li et al. [100] successfully fabricated octadecane@TiO_2_ MEPCMs with *n*-octadecane as the core and titanium dioxide (TiO_2_) as the shell via a sol–gel method. The MEPCMs exhibited a uniform spherical morphology, achieving uniform particle diameters between 2 and 4 μm (Figure 1a), and they demonstrated excellent TES performance, with melting and solidifying enthalpies of 154.24 J/g and 154.26 J/g, respectively, and an encapsulation efficiency of 65.84%. Based on the synthesized MEPCMs, a CNT-enhanced suspension was further developed. As shown in Figure 1b, the suspension containing only MEPCMs changed into a transparent state because of gravity, while the MEPCMs/CNT composite suspension exhibited good dispersion stability after 7 days due to the interweaving of CNTs. In contrast to pure water, the specific heat and thermal conductivity of the MEPCMs/CNT composite suspension (0.2 wt% CNT) were about 43.57% and 34.48% higher than those of pure water, respectively. More importantly, the photothermal conversion efficiency of the MEPCMs/CNT composite suspensions achieved as high as 86.0 ± 1.5% in the simulated sunlight irradiation experiment with a light intensity of about 1 ± 0.03 W/cm^2^ (Figure 1c), showing its significant potential for effectively capturing and storing solar energy.

**Figure 2:**
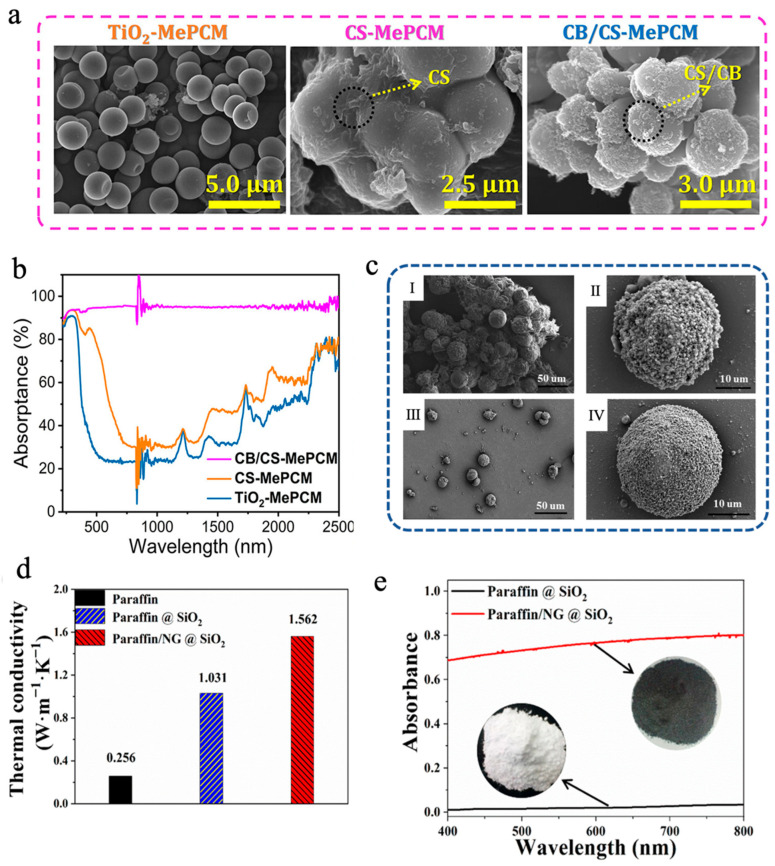
(**a**) SEM images of TiO_2_-MePCM, CS-MePCM, and CB/CS-MePCM [101]. Copyright © 2023, American Chemical Society. (**b**) UV-vis-NIR absorption spectra of CB/CS-MePCM, CS-MePCM, and TiO_2_-MePCM [101]. Copyright © 2023, American Chemical Society. (**c**) SEM images of paraffin@SiO_2_ MEPCMs (I, II) and paraffin/NG@SiO_2_ MEPCMs (III, IV) [102]. Copyright © 2024, Royal Society of Chemistry. (**d**) Thermal conductivities of paraffin/NG@SiO_2_ MEPCMs, paraffin@SiO_2_ MEPCMs, and pure paraffin solid powders [102]. Copyright © 2024, Royal Society of Chemistry. (**e**) UV-vis diffuse reflectance spectra of paraffin/NG@SiO_2_ and paraffin@SiO_2_ MEPCMs [102]. Copyright © 2024, Royal Society of Chemistry.

In addition to simply compounding carbon-based materials with MEPCMs to make suspensions, they can also be introduced during the preparation of microcapsules to fabricate MEPCMs with high photothermal conversion capability. Chen et al. [101] designed a chitosan (CS)/carbon black (CB)-modified phase-change microcapsule (CB/CS-MePCM), employing titanium dioxide (TiO_2_) as an inorganic shell, and encapsulating *n*-docosane as the PCM core, and they then further coated the surface of the microcapsules with a CB/CS composite layer to enhance the solar absorption efficiency. As shown in Figure 2a, TiO_2_-MePCM exhibited a smooth surface and a regular spherical morphology. However, CB/CS-MePCM showed a coarser surface because of the presence of some CB and CS nanoparticles embedded on the surface, which suggested that a CB/CS nanocomposite layer was successfully decorated on TiO_2_-MePCM. The differential scanning calorimetry (DSC) analysis demonstrated that CB/CS-MePCM still retained remarkable TES capacity (>140 J/g), even after undergoing layer-by-layer microencapsulation. Additionally, the leakage rate of CB/CS-MePCM was only 1.73% after heating for 12 h at 65 °C, which was lower than that of CS-MePCM (2.21%) and TiO_2_-MePCM (3.25%), respectively, due to the layer-by-layer microencapsulation. The UV-vis-NIR spectroscopic analysis indicated that the CB/CS-MePCM exhibited excellent light absorption capacity thanks to the reasonable combination of CB and CS (Figure 2b). CB/CS-MePCM achieved 95.04% light absorption efficiency, much higher than that of CS-MePCM (53.94%) and TiO_2_-MePCM (41.60%), respectively. Yuan et al. [102] synthesized paraffin/nano-graphite (NG)@SiO_2_ MEPCMs via in situ polycondensation of tetraethoxysilane (TEOS), forming a SiO_2_ shell to achieve efficient encapsulation of both paraffin and NG. As shown in Figure 2c, compared with paraffin@SiO_2_ MEPCMs, the addition of NG reduced the aggregation of SiO_2_, resulting in paraffin/NG@SiO_2_ MEPCMs with a more complete spherical morphology. The melting and solidification enthalpies of paraffin@SiO_2_ MEPCMs were 91.9 J/g and 89.7 J/g, respectively, while the encapsulation efficiency was determined to be 52.7%. After the introduction of NG, the enthalpy value of paraffin/NG@SiO_2_ MEPCMs decreased slightly, with the encapsulation efficiency dropping to 47.5% due to the partial replacement of paraffin. However, the introduction of NG also led to significant performance enhancements. On the one hand, NG enhanced the thermal conductivity of paraffin/NG@SiO_2_ MEPCMs to 1.562 W m^−1^ K^−1^, approximately 5 times that of pure paraffin (Figure 2d). On the other hand, the introduction of NG also enhanced the absorption capacity of MEPCMs for visible light. Compared with the paraffin@SiO_2_ MEPCMs, the absorption of paraffin/NG@SiO_2_ MEPCMs reached more than 70% (Figure 2e). Incorporating paraffin/NG@SiO_2_ MEPCMs into water enhanced the water’s thermal conductivity, heat capacity, and photothermal performance for solar receiver applications. Through boosting the efficiency of both heat transfer and light absorption, the composite fluid exhibits excellent solar energy capture and storage capabilities, which provides a new material design idea for the performance optimization of solar thermal utilization systems. In addition, Sun et al. [82] utilized graphene oxide (GO) and cellulose nanocrystal (CNC) to construct a stable Pickering emulsion for efficient encapsulation of octadecane (C18) and docosane (C22) through oxidative self-polymerization of dopamine and in situ polymerization MF resin. Polydopamine (PDA) not only had good photothermal conversion ability but also reduced GO to rGO, thereby elevating the solar-to-thermal conversion efficiency of the shell. C22@CNC/rGO/PDA/MF MEPCMs exhibited excellent TES performance and thermal cycling stability, with an encapsulation efficiency for C22 reaching 84.3% and a phase-change enthalpy of 175.4 J/g [82]. After 44 min of simulated light irradiation at 1 W/cm^2^, the temperature of 15 wt% C22@CNC/rGO/PDA/MF MEPCMs slurry increased to 73 °C, higher than that of 15 wt% C22@CNC/GO/MF MEPCMs slurry (55 °C) without PDA and that of 15 wt% C22@CNC/PDA/MF MEPCMs slurry (65 °C) without GO, respectively, indicating a strong synergistic effect of rGO and PDA in boosting photothermal conversion efficiency [82].

### 3.2. Organic Photothermal Materials Integrated with MEPCMs

**Figure 3:**
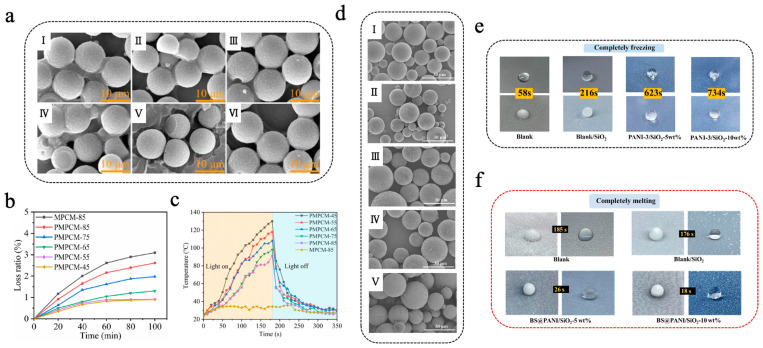
(**a**) SEM images of MPCM-45 (I), MPCM-65 (II), MPCM-85 (III), PMPCM-45 (IV), PMPCM-65 (V), and PMPCM-85 (VI) [105]. Copyright © 2021, Elsevier. (**b**) Loss ratio of MPCM-85 and PMPCMs for 100 min at 80 °C [105]. Copyright © 2021, Elsevier. (**c**) Temperature evolution curves of MPCM-85 and PMPCMs with light on/off [105]. Copyright © 2021, Elsevier. (**d**) SEM images of BS@PANI microcapsules with different aniline concentrations: BS@PEA (I), BS@PANI-1 (II), BS@PANI-2 (III), BS@PANI-3 (IV), and BS@PANI-4 (V) [106]. Copyright © 2024, Elsevier. (**e**) Images of freezing process of water droplets on various coated surfaces at −20 °C [106]. Copyright © 2024, Elsevier. (**f**) Images of melting process of ice on various surfaces when exposed to NIR light irradiation [106]. Copyright © 2024, Elsevier.

Organic photothermal materials typically exhibit high photo-absorption efficiency, photostability, and low preparation cost, establishing platform capabilities for solar–thermal energy harvesting. These materials absorb light energy of characteristic wavelengths through conjugated structures and subsequently transform the light energy into thermal energy via non-radiative transitions, thereby increasing the temperature of the materials [103,104]. Common organic photothermal materials mainly consist of organic small-molecule dyes such as diketopyrrolopyrrole (DPP), indocyanine green (ICG), Prussian blue (PB), etc., and conjugated polymers such as polyaniline (PANI), polydopamine (PDA), polypyrrole (PPY), etc. Yuan et al. [105] prepared PDA@MF/*n*-eicosane microencapsulated phase-change materials (PMPCMs) with a double-layer structure via a two-step in situ polymerization method, in which MF resin was utilized to encapsulate *n*-eicosane to form the inner shell of MPCMs, followed by self-polymerization of dopamine hydrochloride to form a PDA outer shell. As shown in Figure 3a, both the single-layer MPCMs and the double-layer PMPCMs exhibited regular spherical shapes; in particular, the PMPCM-85 (with 85% *n*-eicosane) had a smoother and more uniform surface, indicating that the PDA layer helped enhance the morphological integrity [105]. PMPCM-85 had a significant phase-change enthalpy of 199.4 J/g and demonstrated excellent leakage resistance, with a leakage rate of only 2.6% after a 100 min leakage test at 80 °C (Figure 3b). Moreover, PDA, recognized as an effective photothermal material, significantly enhanced the photothermal conversion efficiency of PMPCMs. As shown in Figure 3c, under simulated solar irradiation, all PCPCM samples rapidly reached more than 80 °C within 160 s, which was much higher than that of the single-layer MPCM-85, which only reached about 34 °C. Similarly, Zhang et al. [106] initiated the polymerization of epoxy sodium p-styrenesulfonate and epoxy acrylate under ultraviolet lamp irradiation, encapsulating butyl stearate (BS) to obtain BS@PEA microcapsules featuring sulfonic acid groups on their surface. Subsequently, aniline was introduced and adhered to the surface of BS@PEA microcapsules through electrostatic interaction, followed by polymerization initiated by an ammonium persulfate aqueous solution. This process thus prepared double-shell multifunctional BS@PANI microcapsules for constructing anti-icing/de-icing composite coatings for all weather conditions [106]. As shown in Figure 3d, the surface of the microcapsules became progressively rougher with the rise in aniline concentration, thereby confirming the successful deposition and shell formation of PANI on BS@PEA microcapsules. The PANI not only improved the integrity of the shell but also imparted remarkable photothermal conversion capability, thermal conductivity, and anti-corrosion functionality. As shown in Figure 3e, compared to the uncoated surface, water droplets solidified approximately 12.6 times slower on the composite coating that included 10 wt% BS@PANI-3 microcapsules (incorporating 15 wt% aniline) on a chiller at –20 °C. Furthermore, under simulated near-infrared (NIR) irradiation (200 mW/cm^2^), ice melting time required only 18 s on the composite coating, whereas the blank coating needed a nearly 10 times longer amount of time (Figure 3f). The introduction of PANI-based photothermal MEPCMs endowed the coating with excellent thermal regulation capability, opening new avenues for the fabrication of multifunctional anti-icing/de-icing coatings.

For the purpose of examining how various surfactants affect the polymerization process and the effectiveness of microcapsules, Li et al. [107] designed PPY-modified yeast phase-change microcapsules (PYPs) to boost the photothermal conversion efficiency and energy storage capacity of yeast phase-change microcapsules (YPs). Three surfactants with distinct hydrophilic and lipophilic balance (HLB) values—sodium dodecylbenzene sulfonate (SDBS), Span 80, and sodium dodecyl sulfate (SDS)—were used to systematically analyze their effects on the synthesis of PPY. The results showed that when SDS was used as the surfactant with a 2:1 molar ratio to pyrrole, the PYPs (SDS) exhibited the best thermal conductivity and the lowest electrical resistivity (1.343 Ω·cm) [107]. This can be attributed to the high HLB of SDS, which facilitated small, stable micelles and a higher degree of polymerization, resulting in a dense and continuous PPY layer. Meanwhile, PYPs (SDS) demonstrated strong absorption of near-infrared light in the 400–1000 nm wavelength range, with a maximum absorbance of 1.19, whereas unmodified capric acid (CA)/lauric acid (LA) showed negligible light absorption in this spectral region. The incorporation of PPY significantly enhanced the photothermal conversion efficiency of CA/LA. In simulated light irradiation experiments, after 600 s of irradiation, PYPs (SDS) reached a maximum temperature of 35 °C, which was 7 °C higher than that of CA/LA [107].

Beyond the single addition of organic photothermal materials, developing organic composite photothermal phase-change microcapsules can achieve more efficient photothermal conversion performance. Li et al. [108] prepared PDA and PPY composite photothermal phase-change microcapsules (SCN@PAP micro-PCMs), with *n*-octadecane (*n*-OD) as the core component and silicon dioxide (SiO_2_) as the shell component, and they then deposited a PDA/PPY composite layer (PAP) on the surface to achieve efficient photothermal conversion. The findings indicated that incorporating PDA can enhance the optical performance of PPY. When the mass ratio of PY to DA was 1:0.032 and that of PAP to SCN micro-PCMs was 1:6, the SCN@PAP micro-PCMs exhibited the best light absorption performance and optimal morphology, achieving a photothermal conversion efficiency of 97.31%. The DSC results indicated that the SCN@PAP micro-PCMs displayed a melting enthalpy of 145.3 J/g, with an enthalpy loss of less than 1 J/g following 100 heating–cooling cycles, showcasing remarkable photothermal conversion efficiency and thermal storage stability.

### 3.3. Metal-Based Photothermal Materials Integrated with MEPCMs

**Figure 4:**
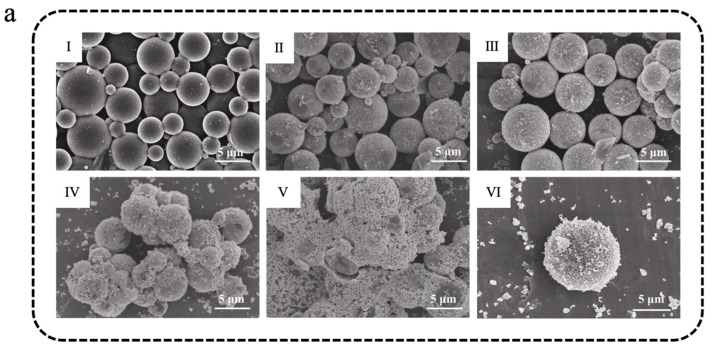

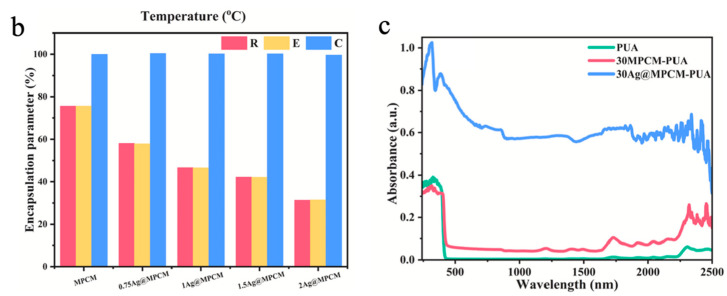
(**a**) SEM images of MPCM (I), 0.75Ag@MPCM (II), 1Ag@MPCM-85 (III,VI), 1.5Ag@MPCM (IV), and 2Ag@MPCM (V) [111]. Copyright © 2025, Elsevier. (**b**) The encapsulation ratio (R), efficiency of encapsulation (E), and efficiency of TES (C) of MPCM and Ag@MPCM [111]. Copyright © 2025, Elsevier. (**c**) Spectra of composite films in the UV-vis-NIR wavelength range [111]. Copyright © 2025, Elsevier.

Metal-based photothermal materials represent another category of efficient photothermal conversion materials, primarily operating through the LSPR effect, which exhibits significant light absorption in the vis-NIR regions. The LSPR effect is defined as the coherent oscillation of free electrons on metal nanoparticle surfaces excited by incident light at their resonant frequency, with subsequent conversion of light energy to heat through non-radiative decay mechanisms [109,110]. Meanwhile, the remarkable thermal conductivity of metal materials helps facilitate the rapid diffusion or directional accumulation of heat inside them, thereby further improving the photothermal conversion efficiency. Common metal-based photothermal materials mainly include nanoparticles of gold (Au), silver (Ag), and copper (Cu), etc. In MEPCM systems, the metal content typically ranges from trace amounts to several tens of weight percent, depending on the roles of the metal, such as functioning as a synergistic modifier and the primary photothermal component. Zhou et al. [111] successfully prepared silver nanoparticles (AgNPs)-modified microencapsulated phase-change materials (Ag@MPCM) and then integrated them into polyurethane acrylates (PUA) via UV curing. Through the LSPR effect, AgNPs enhanced both photothermal conversion and thermal conductivity of the Ag@MPCM. As shown in Figure 4a, with the increase in AgNO_3_ added during the reaction, the surface of the microcapsules gradually became rougher, indicating the effective deposition of AgNPs onto MPCMs (0.75Ag@MPCM, 1Ag@MPCM, 1.5Ag@MPCM, and 2Ag@MPCM, representing AgNO_3_-to-MPCM mass ratios of 0.75:1, 1:1, 1.5:1, and 2:1, respectively). Because AgNPs did not contribute to LHS during the phase-change temperature test, the encapsulation ratio (R) and efficiency of encapsulation (E) of Ag@MPCM gradually decreased with the increase in AgNPs, but the efficiency of TES (C) could still remain as high as 99% (Figure 4b) [111]. After 100 heating–cooling cycles, Ag@MPCM maintained stable thermal cycling performance, especially 1Ag@MPCM, with almost no enthalpy loss. In addition, owing to the high thermal conductivity of AgNPs, 1Ag@MPCM achieved a thermal conductivity of 0.643 W m^−1^ K^−1^, 2.42 times that of the unmodified MPCM. Subsequently, MPCM and 1Ag@MPCM were integrated with flexible PUA resin to produce composite films. In comparison to pure PUA and 30MPCM-PUA (30 wt% MPCM in PUA), the 30Ag@MPCM-PUA sample (30 wt% 1Ag@MPCM in PUA) demonstrated a wider range of light absorption and higher absorption intensity throughout the spectrum (Figure 4c). During the simulated solar irradiation, 30MPCM-PUA showed a clear phase-transition plateau, while 30Ag@MPCM-PUA exhibited the fastest heating rate and reached the highest temperature. Furthermore, the temperatures of both 30Ag@MPCM-PUA and 30MPCM-PUA remained slightly elevated compared with PUA due to the thermal release from the PCM after the simulated light was turned off [111].

**Figure 5:**
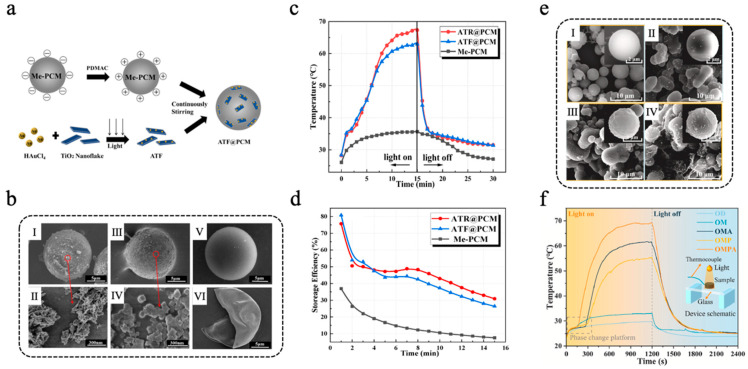
(**a**) Diagram of ATF and ATF@PCM preparation process [112]. Copyright © 2022, Elsevier. (**b**) SEM images of ATR@PCM: (I) overall morphology and (II) magnified view of the area indicated by the red circle, ATF@PCM: (III) overall morphology and (IV) magnified view of the area indicated by the red circle, intact Me-PCM (V), and broken Me-PCM (VI) [112]. Copyright © 2022, Elsevier. (**c**) Temperature–time curves of Me-PCM, ATF@PCM, and ATR@PCM [112]. Copyright © 2022, Elsevier. (**d**) The thermal storage efficiency of ATR@PCM, ATF@PCM, and Me-PCM [112]. Copyright © 2022, Elsevier. (**e**) SEM images of OM (I), OMA (II), OMP (III), and OMPA (IV) [113]. Copyright © 2025, Elsevier. (**f**) Diagrams of photothermal conversion experiment and temperature change curves of OMPA, OMA, OM, and OD [113]. Copyright © 2025, Elsevier.

In more application scenarios, metal-based photothermal materials are often combined with other functional materials to achieve multifunctionality and synergistic enhancement. Peng et al. [112] developed an Au/TiO_2_@PCM composite microcapsule, leveraging the LSPR effect of gold nanoparticles (AuNPs) to improve the visible light absorption of TiO_2_, thereby enabling efficient photocatalytic hydrogen production and TES. AuNPs were loaded onto TiO_2_ nanorods (TNR) and TiO_2_ nanosheets (TNF) via a photo-deposition method, yielding samples denoted as ATR and ATF, respectively. ATF@PCM and ATR@PCM were fabricated through the method of electrostatic self-assembly, with the detailed preparation procedure and microstructures illustrated in Figure 5a,b. After 15 min of simulated light irradiation, the temperatures of ATF@PCM and ATR@PCM reached 63.0 °C and 67.3 °C, respectively, both higher than that of unmodified Me-PCMs (Figure 5c). The thermal storage efficiency of ATR@PCM, ATF@PCM, and Me-PCM is shown in Figure 5d. During photothermal catalytic hydrogen production experiments, the light-to-hydrogen (LTH) efficiency of ATF@PCM and ATR@PCM was 5.0‰ and 11.4‰, respectively, after 3 h of simulated light irradiation, representing 56.1% and 53.8% improvements compared with pure ATF and ATR without microencapsulation. Additionally, ATR@PCM exhibited superior performance in both photothermal conversion and photocatalytic hydrogen production compared with ATF@PCM, which was due to the larger specific surface area of ATR. Lou et al. [113] designed a synergistically enhanced multilayer melamine-formaldehyde resin@octadecane microcapsule (OMPA) integrated with PPY and silver nanoparticles (AgNPs) to improve photothermal conversion efficiency. Octadecane (OD) was used as the core PCM, which was initially encapsulated with an MF resin shell to obtain OM. Subsequently, a PPY layer was applied to the surface of OM through a simple oxidative self-polymerization process to obtain OMP. Finally, AgNPs were deposited onto the surface of OMP through chemical plating to construct a multifunctional composite shell. As shown in Figure 5e, SEM images revealed that a PPY layer improved the surface roughness of the microcapsules, which provided numerous attachment sites for AgNPs. As a result, the AgNPs loading of OMPA was significantly higher than that of OMA (without PPY modification). The DSC results revealed that the enthalpy of melting of OMPA was 101.3 J/g, indicating a high capacity for TES. After 100 heating–cooling cycles, neither the chemical structure nor the thermal properties of OMPA changed significantly, demonstrating excellent cycling stability. The synergistic effect of the LSPR effect of AgNPs and the conjugation effect of PPY significantly improved the photothermal conversion efficiency of OMPA. Additionally, the thermal conductivity of OMPA was boosted because of the electron transfer occurring between AgNPs and PPY. The photothermal energy storage conversion tests and the temperature change curves of OMPA, OMA, OM, and OD are shown in Figure 5f. Analysis indicated that the photothermal conversion efficiency of OMPA was 94.3%, surpassing those of OMP (83.6%) and OMA (88.2%). The thermal conductivity of OMPA attained a value of 1.0343 W m^−1^ K^−1^, representing an increase of 374.2% compared to that of pure OD, thereby promoting the rapid storage of thermal energy converted from solar energy. Similarly, Zhang et al. [114] adhered PDA to the surface of paraffin@silicon dioxide (Pn@SiO_2_) microcapsules via self-polymerization and then utilized the weak reducibility of PDA to reduce silver ammonia ions into AgNPs to form Pn@SiO_2_@PDA/Ag microspheres. The Pn@SiO_2_@PDA/Ag microcapsules had a phase-change enthalpy of more than 130 J/g and a high photothermal conversion efficiency of 88.7%.

### 3.4. Other Photothermal Materials Integrated with MEPCMs

In addition to the photothermal conversion materials mentioned above, MXene, as a popular material in recent years, has also been widely studied and applied. Naguib et al. [115] synthesized titanium carbide (Ti_3_C_2_) MXene by selectively etching the Al layer from Ti_3_AlC_2_ using hydrofluoric acid (HF) at room temperature, followed by sonication in methanol. MXene is a type of graphene-like two-dimensional (2D) transition metal carbide, nitride, or carbonitride, obtained by selectively etching the A layer from M_n+1_AX_n_ phases, where A is a weakly bonded group element, M represents an early transition metal element, and X stands for carbon (C) or nitrogen (N) [116,117,118]. As a result of the LSPR effect, MXene demonstrates exceptional light absorption and photothermal conversion abilities within the vis-NIR spectra. Incorporating MXene into MEPCMs can endow the system with strong thermal storage capacity, high thermal conductivity, and remarkable photothermal conversion performance.

**Figure 6:**
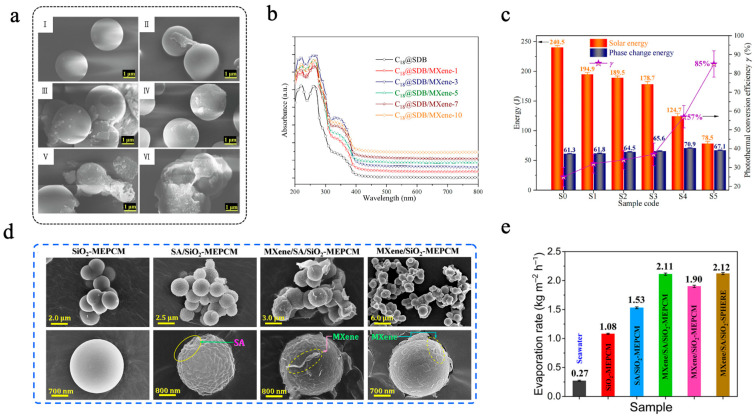
(**a**) SEM images of C_18_@SDB (I), C_18_@SDB/MXene-1 (II), C_18_@SDB/MXene-3 (III), C_18_@SDB/MXene-5 (IV), C_18_@SDB/MXene-7 (V), and C_18_@SDB/MXene-10 (VI) [119]. Copyright © 2023, Elsevier. (**b**) UV-vis-NIR absorption spectra [119]. Copyright © 2023, Elsevier. (**c**) Solar-to-heat conversion efficiency and energy storage performance of the microcapsules [119]. Copyright © 2023, Elsevier. (**d**) SEM images of SiO_2_-MEPCMs, SA/SiO_2_-MEPCMs, MXene/SA/SiO_2_-MEPCMs, and MXene/SiO_2_-MEPCMs [120]. Copyright © 2023, Elsevier. (**e**) Evaporation rates of seawater and evaporators based on microcapsule samples [120]. Copyright © 2023, Elsevier.

Zhao et al. [119] used Ti_3_C_2_ MXene nanosheets to modify phase-change microcapsules. The C_18_@SDB/MXene consisted of styrene divinylbenzene (SDB) copolymer as the shell, *n*-octadecane as the core, and Ti_3_C_2_ MXene nanosheets were anchored onto the surface of microcapsules via hydrogen bonding interactions with the emulsifier polyvinylpyrrolidone (PVP) [119]. The images depicting the microstructure of six distinct types of microcapsules, each containing varying amounts of Ti_3_C_2_ MXene, are presented in Figure 6a. Compared with the C_18_@SDB, some thin slices can be observed on the surface of C_18_@SDB/MXene, suggesting that the SDB/MXene composite shell was successfully synthesized. When the Ti_3_C_2_ MXene content reached 0.67 wt% (C_18_@SDB/MXene-10), the ability of the microcapsules to disperse had been substantially reduced, suggesting that further addition of Ti_3_C_2_ MXene was impractical. In simulated light irradiation experiments, the light absorption performance of C_18_@SDB/MXene was significantly improved in the 200–800 nm solar spectrum compared to C_18_@SDB (Figure 6b). As shown in Figure 6c, the photothermal conversion efficiency (γ) of microcapsules gradually increased with the increase in Ti_3_C_2_ MXene, and the γ value of the C_18_@SDB/MXene-10 reached 85 ± 7%, representing a 240% enhancement compared to that of C_18_@SDB. Similarly, Zhang et al. [120] constructed a multilayer phase-change microcapsule (MXene/SA/SiO_2_-MEPCMs) for sustainable solar-driven seawater desalination. In this design, *n*-docosane was first encapsulated with a silica (SiO_2_) shell to form SiO_2_-MEPCMs. A sodium alginate (SA) coating was then introduced via a crosslinking reaction to form SA/SiO_2_-MEPCMs. Finally, MXene nanosheets were anchored onto the surface through hydrogen bonding, resulting in the final MXene/SA/SiO_2_-MEPCMs structure [120]. SEM images of the obtained MEPCMs are shown in Figure 6d. The surface of SA/SiO_2_-MEPCMs was significantly rougher than that of SiO_2_-MEPCMs. After MXene nanosheets modification, both MXene/SA/SiO_2_-MEPCMs and MXene/SiO_2_-MEPCMs exhibited a few thin slices, indicating the successful deposition of MXene. DSC results showed that MXene/SA/SiO_2_-MEPCMs possessed high latent thermal capacity, with a crystallization enthalpy (Δ*H*_c_) and a melting enthalpy (Δ*H*_m_) of 151.7 J/g and 156.1 J/g, respectively. The encapsulation efficiency exceeded 60%, and the leakage rate after 24 h of heating was only 0.8 wt%. Thanks to the excellent photothermal conversion capabilities and light absorption in the vis-NIR regions, the light absorption efficiency of MXene/SA/SiO_2_-MEPCMs reached as high as 97.2%, significantly higher than that of SiO_2_-MEPCMs (6.4%) and SA/SiO_2_-MEPCMs (77.7%). In solar-driven desalination experiments, MXene/SA/SiO_2_-MEPCMs exhibited outstanding interfacial evaporation performance, achieving an evaporation rate of 2.11 kg m^−2^ h^−1^ (Figure 6e). Moreover, compared to MXene/SA/SiO_2_-SPHERE (without PCM), the cumulative evaporation mass after five one-sun illumination and natural cooling cycles increased by 1.4 kg m^−2^, enabling continuous seawater desalination under intermittent solar irradiation [120].

To create inorganic hydrated salt microcapsules that possess outstanding photothermal conversion efficiency and heat storage capacity, Lu et al. [121] designed and prepared composite phase-change microcapsules (MXene/MPCMs), in which Na_2_HPO_4_·12H_2_O was employed as the phase-change core. The SiO_2_ shell was formed via the hydrolysis of TEOS, and then Ti_3_C_2_ MXene was incorporated via high-speed stirring. The composite structure of MXene and SiO_2_ significantly enhanced the photothermal responsiveness of MPCMs. In the simulated light irradiation experiment with an irradiance of 100 mW/cm^2^, both MPCM-1 (prepared by the mixer) and MPCM-2 (prepared by shearing mechanism) exhibited increased heating rates and elevated maximum temperatures with the addition of Ti_3_C_2_ MXene, especially MPCM-2 [121]. Meanwhile, the melting enthalpy of MPCM-1 and MPCM-2 was 169.62 J/g and 115.4 J/g, respectively, which highlights their promising application potential in solar energy harvesting and conversion systems [121].

In summary, the incorporation of photothermal materials greatly enhances the solar absorption and thermal conductivity of MEPCMs, thereby improving their photothermal conversion efficiency. By converting absorbed solar energy into latent heat through the phase-change process, these MEPCMs achieve efficient energy capture and storage, expanding their potential in solar energy utilization and anti-icing applications.

However, carbon-based photothermal agents often suffer from poor dispersion and weak interfacial compatibility within the polymer matrix. These issues can lead to particle aggregation and reduced photothermal uniformity. Organic photothermal materials are susceptible to photodegradation and thermal instability under prolonged irradiation. Metallic photothermal additives provide excellent conductivity but are constrained by high cost, oxidation susceptibility, and relatively heavy density. Collectively, these drawbacks limit the long-term stability, cycling durability, and energy storage density of photothermal MEPCMs. Future research should therefore focus on improving the dispersion and interfacial bonding of carbon-based additives through surface modification or functionalized encapsulation techniques. It is also necessary to enhance the UV and oxidation resistance of organic and metallic photothermal agents via hybridization or coating strategies. Optimizing capsule architectures to balance photothermal efficiency and latent heat capacity will be equally important. In addition, more efforts are needed to develop cost-effective, environmentally friendly, and scalable synthesis methods to promote the industrial implementation of photothermal MEPCMs in real-world systems such as solar thermal storage, smart coatings, and thermal regulation textiles.

## 4. Photocatalytic MEPCMs

Photocatalytic materials represent a category of functional substances that can absorb solar energy and form electron–hole pairs to facilitate redox reactions. When the photon energy that photocatalytic materials absorb exceeds their bandgap energy, electrons (e^−^) in the valence band (VB) are excited to the conduction band (CB), forming electron–hole pairs (e^−^/h^+^). Subsequently, the e^−^ and h^+^ migrate to the semiconductor surface, where e^−^ can reduce acceptors and h^+^ can oxidize donors. The resulting reactive oxygen species (ROS) can further degrade pollutants, split water for hydrogen production, or reduce CO_2_, achieving the purpose of the photocatalytic reaction [85,88,122,123,124]. Common photocatalytic materials mainly include ZnO, TiO_2_, Cu_2_O, MoS_2_, CdS, etc. The combination of photocatalytic materials with MEPCMs can achieve the synergistic optimization of light energy conversion, TES, and photocatalytic functions, enhancing light energy utilization efficiency while promoting the stability of photocatalytic reactions.

**Figure 7:**
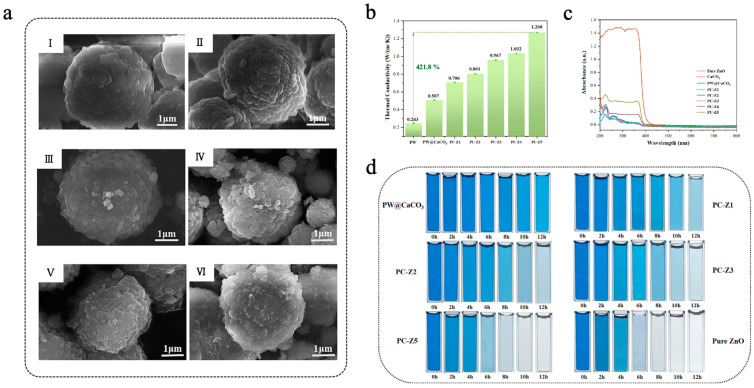
(**a**) SEM images of PW@CaCO_3_ (I), PC-Z1 (II), PC-Z2 (III), PC-Z3 (IV), PC-Z4 (V), and PC-Z5 (VI) [124]. Copyright © 2024, Elsevier. (**b**) Thermal conductivity of pure ZnO, CaCO_3_, PW@CaCO_3_, PC-Z1, PC-Z2, PC-Z3, PC-Z4, and PC-Z5 [124]. Copyright © 2024, Elsevier. (**c**) UV-vis spectra of pure ZnO, CaCO_3_, PW@CaCO_3_, PC-Z1, PC-Z2, PC-Z3, PC-Z4, and PC-Z5 [124]. Copyright © 2024, Elsevier. (**d**) Digital images of photocatalytic degradation of MB dye solutions with various catalysts [124]. Copyright © 2024, Elsevier.

Due to its wide bandgap (~3.4 eV), nano-ZnO exhibits excellent photocatalytic activity in the ultraviolet (UV) region [122,123]. Chen et al. [124] fabricated PW@CaCO_3_/ZnO microcapsules via an in situ polymerization method, which used paraffin wax (PW) as the core material, calcium carbonate (CaCO_3_) as the shell material, and doped ZnO nanoparticles with different mass fractions (0.03–0.15 wt%) onto the surface of the shell to achieve dual applications of TES and photocatalytic degradation. As shown in Figure 7a, the DSC findings indicated that the phase-change enthalpy of PW@CaCO_3_/ZnO microcapsules initially increased with the increase in ZnO addition, followed by a decrease possibly due to emulsion destabilization introduced by excess ZnO nanoparticles. Among all samples, PC-Z1 (with a ZnO content of 0.03 wt%) had the highest phase-change enthalpy, with a melting enthalpy of 93.1 J/g, which was significantly higher than that of PW@CaCO_3_ (51.9 J/g).Concurrently, PW@CaCO_3_/ZnO microcapsules exhibited remarkable thermal stability and high thermal conductivity during the phase-change process, and PC-Z5 (the content of ZnO was 0.15 wt%) achieved a maximum thermal conductivity of 1.268 W/(m·k), which was 150.1% higher than that of PW@CaCO_3_ (Figure 7b). As shown in Figure 7c, UV-vis spectroscopy showed that the light absorption capacity of PW@CaCO_3_/ZnO microcapsules was enhanced with the increase in ZnO content. The photocatalytic efficiency was evaluated by dispersing the microcapsules in a methylene blue (MB) dye solution. As shown in Figure 7d, the photocatalytic efficiency of MB under UV irradiation improved with the increase in ZnO content in the microcapsules. Among all samples, PC-Z5 had the highest photocatalytic efficiency, achieving a degradation rate of 98.97% after 12 h of UV irradiation. These results highlight the great potential of ZnO-modified microcapsules in TES and the mitigation of environmental pollution. As ZnO possesses a relatively wide bandgap (~3.4 eV), its photocatalytic response is mainly restricted to the UV region. To enhance visible-light activity, several modification strategies have been developed, including metal or non-metal doping (e.g., Ag, Mn, F, N, S) [125,126,127,128], construction of heterojunctions [129,130], or coupling carbon materials [131,132]. Yuan et al. [133] employed Ag to improve the visible-light absorption of ZnO by utilizing its plasmonic resonance effect and prepared CS/ZnO-Ag composite microcapsules (SCSM). Under xenon lamp irradiation, SCSM showed a much faster heating rate than microcapsules containing only Ag or ZnO, demonstrating a synergistic effect between the two components. The incorporation of Ag nanoparticles introduces impurity levels and localized surface plasmon resonance, which broaden the absorption range of ZnO from the UV to the visible region.

**Figure 8:**
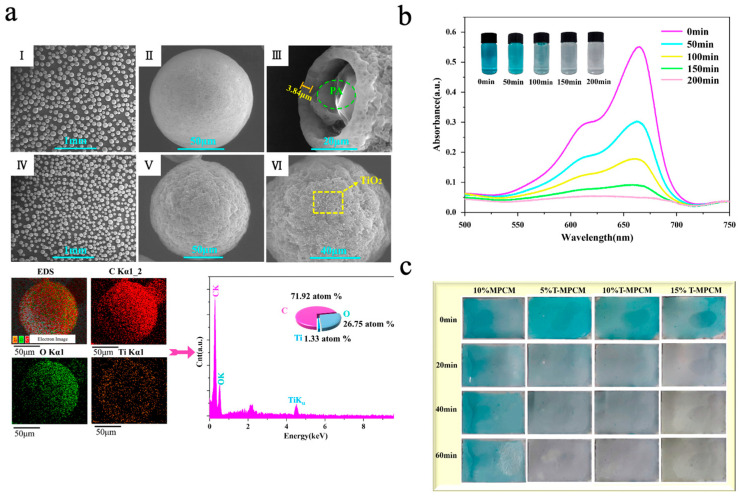
(**a**) SEM images of MPCM (I–III) and T-MPCM (IV–VI) and EDS image of T-MPCM [77]. Copyright © 2024, Elsevier. (**b**) UV-vis spectra of and photos of MB solution [77]. Copyright © 2024, Elsevier. (**c**) Photos of the coatings under UV irradiation [77]. Copyright © 2024, Elsevier.

TiO_2_, as one of the most representative photocatalytic materials, has been widely studied due to its low cost and high chemical stability [134,135]. Zhao et al. [136] prepared TiO_2_-doped SDB copolymer microcapsules via suspension polymerization. To improve compatibility with the organic shell, TiO_2_ nanoparticles were rendered amphiphilic by grafting with 3-glycidoxypropyltrimethoxysilane (KH560), thereby enhancing their anchoring ability at the oil–water interface. DSC results showed that sample S4 (doped with 4.0 wt% TiO_2_) had the best TES performance among all modified samples, with microencapsulation efficiency (E*_en_*) and heat storage efficiency (E*_es_*) reaching 48.0 ± 0.4% and 47.7 ± 0.3%, respectively. However, when the doping rate of TiO_2_ was increased to 5.0 wt% (sample S5), the TES performance dropped significantly, with E*_en_* and E*_es_* decreasing to 30.0 ± 0.7% and 29.8 ± 0.5%, respectively. This degradation may be attributed to excess TiO_2_ nanoparticles disrupting emulsion stability, leading to microcapsule agglomeration and *n*-octadecane leakage. The photocatalytic performance was assessed through the degradation of methylene blue (MB) dye solution under UV irradiation. The degradation rate of MB by sample S4 was 90.92%, while that of sample S0 was only 6.2%. Even after five consecutive photocatalytic cycles, the degradation rate of sample S4 only decreased by 1.36%, indicating good thermal storage performance and efficient photocatalytic property. Similarly, Wang et al. [77] prepared TiO_2_-hybridized phase-change microcapsules (T-MPCM) via a combined approach of solvent evaporation and electrostatic self-assembly using paraffin (PA) as the phase-change core and ethyl cellulose (EC) as the shell material. Due to the positively charged surface of TiO_2_ nanoparticles and the negatively charged nature of EC, the TiO_2_ nanoparticles were uniformly loaded onto the surface of microcapsules through electrostatic attraction without additional coupling agents. As shown in Figure 8a, the microcapsules exhibited good dispersibility and complete spherical structures. The introduction of TiO_2_ nanoparticles increased surface roughness, and the cracked microcapsules also revealed the successful encapsulation of PA. The EDS diagram also identified the presence of Ti, confirming that the nanoparticles present on the microcapsule surface were titanium dioxide. The photocatalytic performance was assessed through the degradation of MB solution under UV-light irradiation. As shown in Figure 8b, the absorbance of MB at 616 nm and 665 nm gradually decreased with prolonged UV irradiation. After 200 min, the MB solution became nearly transparent, with a degradation rate of 91.9%. Even after five consecutive photocatalytic cycles, the degradation rate decreased by only 2.5%, demonstrating excellent photocatalytic performance and good cycling stability. Subsequently, the microcapsules were combined with waterborne acrylic emulsion with varying dosages to prepare composite coatings with photocatalytic functions. As shown in Figure 8c, coatings incorporating T-MPCMs exhibited stronger MB degradation under UV light compared to the MPCM coating, with the photocatalytic effect intensifying as the T-MPCM content increased.

For the purpose of further enhancing the photocatalytic activity of TiO_2_, Zhao et al. [137] developed multifunctional fluorine-doped TiO_2_ phase-change material microcapsules. The introduction of F effectively modulated the band structure of TiO_2_ nanoparticles, broadened their light absorption range, and consequently improved the photocatalytic performance of the microcapsules. The photoluminescence (PL) test results showed that the TiO_2_ nanoparticles exhibited a strong luminescence peak at 390 nm, while the F-doped TiO_2_ PCM microcapsules had almost no luminescence response in this range, indicating that F doping significantly suppressed the radiative recombination process between photogenerated electrons and holes in TiO_2_ nanoparticles, thereby enhancing their photocatalytic efficiency. The photocatalytic performance was assessed via the visible-light-driven degradation of Rhodamine B (RhB) solution (0.025 g/L). The results showed that F-doped TiO_2_ PCM microcapsules exhibited a significantly higher degradation rate than TiO_2_ nanoparticles throughout the illumination period, achieving complete degradation of RhB within 7 h. In addition, the particle size of the microcapsules had a significant effect on their photocatalytic performance. F-doped TiO_2_ PCM microcapsules with a small diameter (100 μm) exhibited a higher specific surface area and greater adsorption capacity, and their reaction constant was seven times greater than that of microcapsules with a diameter of 300 μm, reflecting the important role of size regulation in improving catalytic efficiency.

In addition to the common photocatalytic materials, FeOOH has also been widely studied in recent years as a visible-light-driven photocatalytic material with a wide bandgap and broad photo-response region [138,139]. Xu et al. [140] developed a novel phase-change microcapsule (paraffin@SiO_2_/FeOOH) with SiO_2_/FeOOH as a double-layer shell, which integrated both TES and photocatalytic degradation functionalities. Firstly, paraffin@SiO_2_ microcapsules were constructed by interfacial polymerization, and then the negative charge of the SiO_2_ shell under alkaline conditions was used to attract Fe^2+^ to aggregate and generate FeOOH on its surface in situ, finally obtaining paraffin@SiO_2_/FeOOH microcapsules [140]. SEM and TEM images revealed that the paraffin@SiO_2_ microcapsules exhibited uniform, smooth, and compact spherical structures, while paraffin@SiO_2_/FeOOH microcapsules had rough flake structures on the surface, proving the successful deposition of FeOOH. DSC findings indicated that the melting and freezing enthalpy of paraffin@SiO_2_/FeOOH microcapsules were 104.44 J/g and 101.69 J/g, respectively, with a paraffin encapsulation efficiency of 49.68%, demonstrating good latent heat storage capability. The photocatalytic performance of the microcapsules was evaluated by monitoring the degradation rate of MB solution under natural light. The paraffin@SiO_2_/FeOOH microcapsules achieved a degradation efficiency of 80% within 2 h and reached 94% after 4 h. In cyclic photocatalytic tests, the microcapsules retained stable catalytic activity after five cycles. However, a notable decline in degradation efficiency was observed after ten cycles, which was mainly attributed to partial corrosion and deactivation of FeOOH on the shell. Therefore, the FeOOH-modified microcapsules have good photocatalytic ability, but their long-term stability still requires further improvement.

Moreover, emerging photocatalytic materials, such as perovskites [141,142,143,144], graphitic carbon nitride (g-C_3_N_4_) [145,146], and covalent organic frameworks (COFs) [147,148,149], possess broad light absorption ranges, high charge carrier mobility, large specific surface areas, and tunable band structures, demonstrating significant potential in photocatalysis and solar energy utilization. However, integrating these materials with MEPCM systems faces multiple challenges. Key obstacles include intrinsic stability issues (e.g., halide perovskites degrading under water, heat, or light), complex synthesis procedures incompatible with microencapsulation, and mismatches between optoelectronic properties and the thermal–physical requirements of MEPCMs systems. Additional challenges include interfacial side reactions, structural degradation during long-term cycling, localized overheating causing damage to encapsulation materials, high costs limiting scalability, and inefficient energy transfer pathways resulting in suboptimal photothermal conversion efficiency. Addressing these challenges requires careful material modification, composite structure design, and interfacial engineering.

In short, the incorporation of materials with photocatalytic properties (such as ZnO, TiO_2_, etc.) into MEPCMs not only endows them with the capability to drive catalytic reactions under light irradiation but also achieves the synergistic function of TES and photocatalysis. Photocatalytic MEPCMs demonstrate broad application prospects in fields such as organic pollution degradation and solar-driven hydrogen production.

However, several challenges remain. Most photocatalytic fillers exhibit poor interfacial compatibility and uneven dispersion within the capsule matrix, which may reduce light utilization and catalytic efficiency. In addition, the photocatalytic activity of semiconductor materials often decreases after long-term irradiation due to particle agglomeration or photocorrosion. The relatively high loading of photocatalysts required to achieve strong catalytic effects can also compromise the latent heat capacity of the MEPCM system. Future research should focus on improving the interfacial bonding and dispersion of photocatalysts through surface functionalization or core–shell structural design. Enhancing the stability of semiconductor photocatalysts by doping, heterojunction engineering, or coating strategies is also essential. Furthermore, more attention should be given to developing cost-effective, environmentally friendly, and scalable fabrication methods to promote the practical deployment of photocatalytic MEPCMs in environmental purification and solar-fuel generation systems.

## 5. Photoluminescence MEPCMs

Fluorescence is a typical photoluminescence phenomenon, essentially a non-thermal radiation (cold emission) process [150]. When fluorescence materials are excited by an external light source (such as ultraviolet or visible light), the energy provided by the photons promotes electrons from the lowest energy state to higher energy levels, such as the first or second excited states. Nevertheless, these excited states exhibit instability, causing the electrons to swiftly revert to the ground state via radiative transitions, releasing excess energy in the form of light and thus generating fluorescence [151,152]. Common fluorescent materials include inorganic fluorescent materials (rare earth materials, carbon quantum dots, etc.) and organic fluorescent materials (coumarin, polymer materials, and small-molecule materials, etc.). By incorporating fluorescent materials into the shell or core of MEPCMs, photoluminescence MEPCMs with both fluorescent properties and thermal storage capabilities can be fabricated.

**Figure 9:**
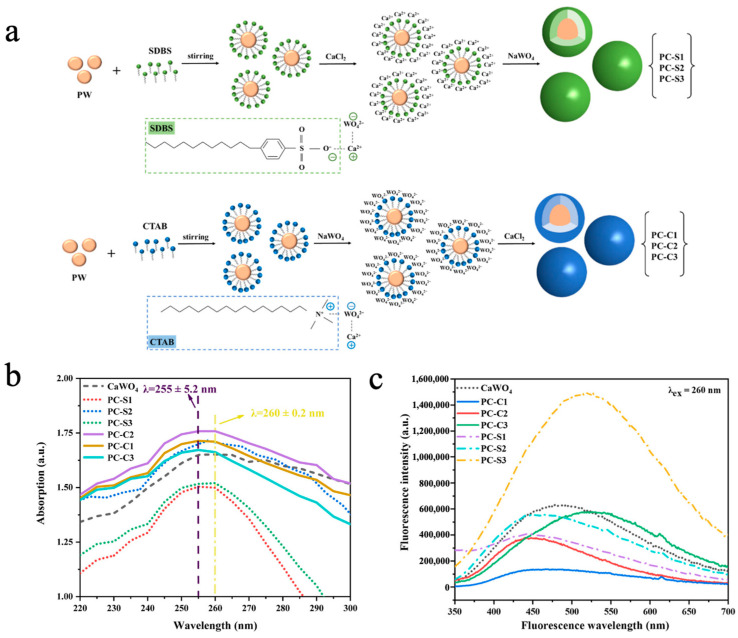
(**a**) Diagram of the PW@CaWO_4_ microcapsules synthesis process [154]. Copyright © 2023, American Chemical Society. (**b**) UV absorption spectra of the PW@CaWO_4_ microcapsules and the CaWO_4_ [154]. Copyright © 2023, American Chemical Society. (**c**) Fluorescence spectra of the PW@CaWO_4_ microcapsules and the CaWO_4_ [154]. Copyright © 2023, American Chemical Society.

Inorganic fluorescent materials such as calcium tungstate (CaWO_4_), zinc tungstate (ZnWO_4_), and copper tungstate (CuWO_4_) exhibit excellent photoluminescence properties due to their unique WO_4_^2−^ tetrahedral structure [153]. Liu et al. [154] prepared multifunctional phase-change microcapsules (PW@CaWO_4_) via an in situ polymerization method using inorganic CaWO_4_ as the shell material and paraffin wax (PW) as the phase-change core. The impact of two distinct emulsifiers, namely, a cationic surfactant, cetyl trimethyl ammonium bromide (CTAB), and an anionic surfactant, sodium dodecyl-benzenesulfonate (SDBS), on the morphology and performance of the MEPCMs was systematically studied. As shown in Figure 9a, the O^2−^ in the hydrophilic group of SDBS formed a covalent bond with Ca^2+^, followed by the reaction of WO_4_^2−^ with Ca^2+^ to generate the CaWO_4_ shell. In contrast, the N^+^ groups in the hydrophilic group of CTAB formed a covalent bond with WO_4_^2−^, followed by the reaction of Ca^2+^ with WO_4_^2−^ to generate the CaWO_4_ shell. SEM images revealed that the sample with SDBS as the emulsifier and a 1:1 core-to-shell ratio (PC-S2) exhibited the optimal spherical morphology, as the opposite charge of SDBS to Ca^2+^ facilitated uniform CaWO_4_ deposition on the PW surface. Because of the CaWO_4_ shell, the PW@CaWO_4_ microcapsules had good thermal conductivity, with a minimum supercooling degree of 1.00 ± 0.08 °C, 3.41 °C lower than that of PW. UV-vis spectroscopy revealed that microcapsules prepared with CTAB (PC-C1/2/3) exhibited stronger UV absorption, accompanied via a minor blue shift in the absorption spectrum due to enhanced oxidation capacity of O^2−^ caused by WO_4_^2−^ attraction to cationic emulsifiers (Figure 9b). As shown in Figure 9c, owing to high sphericity and good encapsulation, the microcapsules prepared with SDBS (PC-S1/2/3) displayed stronger overall fluorescence intensity than those prepared with CTAB (PC-C1/2/3). Furthermore, as the CaWO_4_ shell content increased, fluorescence emission was significantly enhanced and gradually shifted to green, which was attributed to the quantum size effect of the CaWO_4_ shell. Cheng et al. [73] synthesized phase-change microcapsules (NePCMs) with high thermal conductivity, latent heat values, and stability by using zinc tungstate (ZnWO_4_) as the shell material to encapsulate stearic acid (SA) via in situ polymerization. When SDBS was used as the emulsifier and the core-to-shell ratio was 1:1, the NePCMs exhibited uniform and regular spherical morphology, along with the highest phase-change enthalpy of 83.63 J/g. In UV absorption and photoluminescence spectroscopy measurements, NePCMs demonstrated favorable UV absorption capacity and photoluminescent properties. Additionally, the preparation of ZnWO_4_ via this method can reduce the cost by 40%, showing outstanding potential in coating, fluorescent architecture, and textile industries.

**Figure 10:**
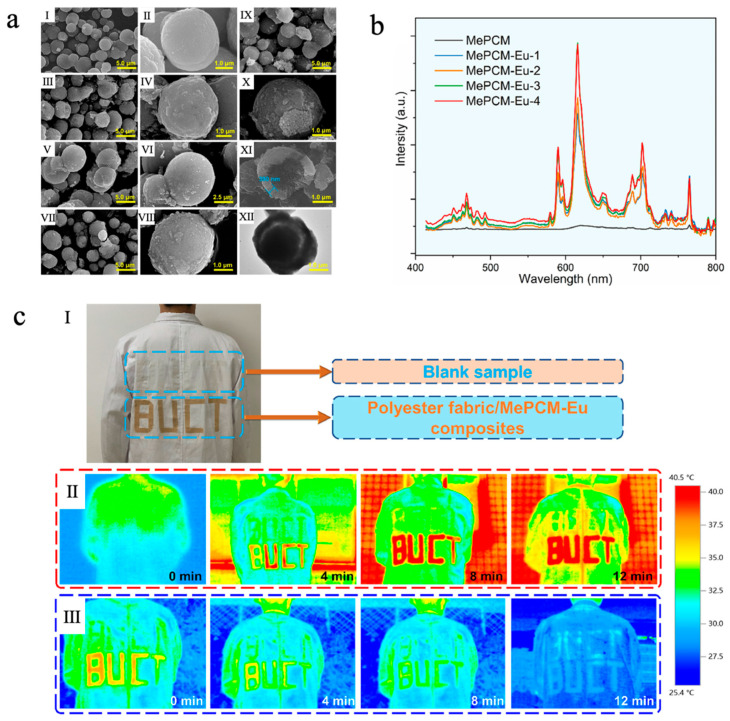
(**a**) SEM images of MePCM (I, II), MePCM-Eu-1 (III, IV), MePCM-Eu-2 (V, VI), MePCM-Eu-3 (VII, VIII), and MePCM-Eu-4 (IX–XI); TEM image of MePCM-Eu-4 (XII) [157]. Copyright © 2022, Elsevier. (**b**) Emission spectra of MePCM-Eu with varying Eu^3+^ doping concentrations under an excitation wavelength of 394 nm [157]. Copyright © 2022, Elsevier. (**c**) An image of a subject wearing a white coat coated with the polyester fabric/MePCM-Eu-2 composite bearing a ‘BUCT’ design, accompanied by pristine polyester fabric as a blank sample (I), infrared thermal images of the white coat captured outdoors under varying solar irradiation durations (II), and those captured indoors under varying natural cooling durations (III) [157]. Copyright © 2022, Elsevier.

In addition, the incorporation of rare earth elements such as ytterbium (Yb) [155], cerium (Ce) [156], europium (Eu) [157], etc., can also endow phase-change microcapsules with desirable photoluminescent properties. Liu et al. [157] fabricated a europium-doped photoluminescent phase-change microcapsule (MePCM-Eu) via a Pickering emulsion templating method, which consisted of *n*-docosane as the core and a CaCO_3_/Fe_3_O_4_ composite as the shell. CaCO_3_ was used as the inorganic shell material due to its low cost, excellent thermal stability, and support for fluorescence emission, while the introduction of Eu^3+^ ions significantly enhanced the luminescence intensity. Fe_3_O_4_ nanoparticles, serving as both emulsion stabilizers and photothermal agents, not only improved the solar–thermal conversion efficiency but also reinforced the structural stability of the shell. As shown in Figure 10a, with increasing Eu^3+^ doping concentration, the surface of the microcapsules gradually became rough, but they still maintained a regular, compact core–shell spherical morphology, indicating good encapsulation integrity. Meanwhile, all MePCM-Eu samples exhibited phase-change enthalpies exceeding 125 J/g, demonstrating outstanding TES capability. The thermal responsiveness and thermal conductivity of MePCM-Eu-2 (doped with 0.134 g of Eu(NO_3_)_3_·6H_2_O) were improved thanks to the addition of Fe_3_O_4_ nanoparticles, which had good photothermal conversion capacity and high thermal conductivity. Photoluminescence measurements showed that MePCM-Eu exhibited Eu^3+^ emission peaks at 468, 589, 616, 702, and 765 nm under 394 nm excitation, while no significant emission was observed in MePCM (without Eu^3+^ doping), confirming that Eu^3+^ doping markedly enhanced luminescence intensity (Figure 10b). In the application test, MePCM-Eu-2 was loaded onto polyester fabric and cut into the shape of ‘BUCT’ and finally adhered to a white coat. As shown in Figure 10c, it effectively absorbed and stored solar thermal energy during sunlight irradiation and gradually released heat at natural cooling to improve physiological comfort. When under UV illumination, the coating displayed a distinct red fluorescent pattern, indicating potential for visual recognition and smart textile applications.

**Figure 11:**
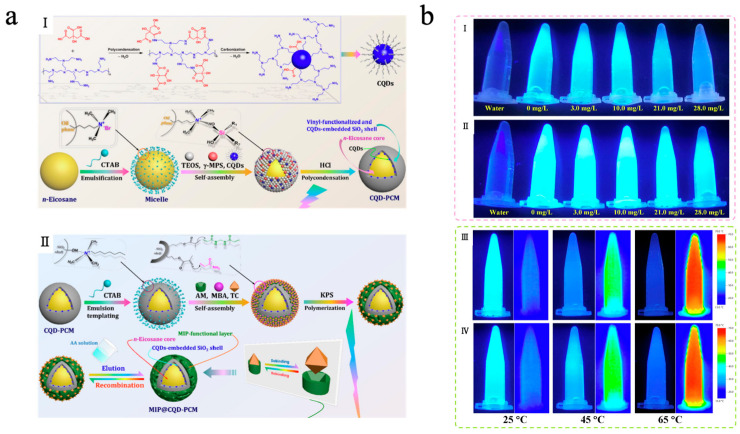
(**a**) Schematic of CQDs and CQD-PCM synthesis (I); schematic of MIP@CQD-PCM synthesis (II) [160]. Copyright © 2021, Elsevier. (**b**) Digital images of MIP@CQD-PCM (I) and NIP@CQD-PCM (II) suspensions under 365 nm UV irradiation at varying tetracycline concentrations. Digital images (**left**) and infrared thermograms (**right**) of MIP@CQD-PCM (III) and MIP@CQD-SPR (IV) suspensions under 365 nm UV irradiation at different equilibrium temperatures [160]. Copyright © 2021, Elsevier.

With the deepening research fluorescence regulation mechanisms, the fluorescence phenomenon not only depends on the energy level structure of molecules but is also significantly influenced by their aggregation state and the surrounding environment. Traditional luminescent molecules typically exhibit efficient fluorescence in dilute solutions; however, their fluorescence intensity is often weakened or even completely quenched in concentrated solutions or aggregated states. This phenomenon is known as aggregation-caused quenching (ACQ). By leveraging this phenomenon, integrating luminescent molecules with MEPCMs can achieve not only TES and temperature regulation but also reversible regulation of fluorescence [158,159]. Yu et al. [160] designed a fluorescence sensing system (MIP@CQD-PCM) by integrating molecularly imprinted MEPCMs with carbon quantum dots (CQDs) for the efficient detection and recognition of tetracycline. As shown in Figure 11a, in this system, *n*-eicosane served as the phase-change material core, while CQDs were embedded within a SiO_2_ shell. The SiO_2_ shell surface was coated with a tetracycline-templated molecularly imprinted polymer (MIP) layer, after which the tetracycline template was eluted from the MIP layer. By combining the molecular imprinting technique with specific recognition sites and the fluorescent properties of CQDs, the system exhibited high adsorption of tetracycline with a fluorescence quenching response via ACQ. The MIP@CQD-PCM exhibited excellent TES capacity and stable phase transition cycling behavior, with an E*_en_* of 77.11% and an E*_es_* of 76.74%, which enhanced the accuracy and efficiency of tetracycline detection under high-temperature conditions. Furthermore, as shown in Figure 11b, the system exhibited a clear linear relationship between tetracycline concentration and fluorescence quenching intensity, in contrast to NIP@CQD-PCM without an MIP template. Due to the PCM core, the system showed better temperature-controlled fluorescence recognition performance compared to MIP@CQD-SPR without *n*-eicosane in high-temperature environments.

Therefore, the introduction of luminescent materials, such as rare earth materials, inorganic fluorescent materials, or CQDs, enables the fabrication of MEPCMs with both TES and photoluminescence functions. Fluorescent MEPCMs not only exhibit excellent thermal response characteristics but also generate stable fluorescence under specific wavelength excitation, expanding the application prospects in fields such as fluorescent architecture, smart coatings, and environmental detection.

However, some challenges remain to be addressed. The luminescence intensity of rare earth and fluorescent materials can be significantly affected by the thermal cycling process, leading to gradual fluorescence attenuation. In addition, the compatibility between luminescent fillers and the polymer shell is often limited, which may result in aggregation or fluorescence quenching. The relatively complex synthesis routes and high cost of rare earth materials also hinder their large-scale application. Future research should therefore focus on improving the interfacial stability between luminescent fillers and the encapsulation matrix, as well as enhancing the photostability of fluorescent agents under repeated heating and cooling cycles. Developing low-cost, high-efficiency luminescent alternatives—such as surface-functionalized CQDs or hybrid nanostructures—could further improve performance. Moreover, efforts should be directed toward establishing scalable and environmentally sustainable synthesis techniques to promote the integration of luminescent MEPCMs into practical applications, including building illumination, smart coatings, and environmental monitoring.

## 6. Conclusions and Outlook

In summary, this article systematically reviews the recent research progress in photo-responsive MEPCMs, with a focus on the integration and performance optimization of three major types of functional materials: photothermal conversion, photocatalysis, and photoluminescence. The incorporation of photothermal materials significantly enhances the solar absorption capacity and thermal conductivity of MEPCMs, enabling efficient solar-to-thermal energy conversion and storage. The integration of photocatalytic materials allows MEPCMs to drive chemical reactions under light irradiation, allowing for the simultaneous realization of TES alongside functions like organic pollutant degradation and solar-driven hydrogen production. The introduction of luminescent components, such as rare earth materials, inorganic fluorescent materials, or carbon quantum dots, imparts stable photoluminescent properties to MEPCMs, thereby promoting the applications of MEPCMs in fluorescent architecture, smart coatings, and environmental sensing.

Despite significant progress in the development of photo-responsive MEPCMs, several key challenges remain to be addressed:

1. Trade-off between functional material loading and energy storage performance. Although photo-responsive components enhance light responsiveness, they do not directly contribute to the phase-change process. Excessive loading will significantly reduce the overall energy storage density.
2. High cost and complex fabrication processes. Most photo-responsive materials currently employed are expensive and require delicate synthesis and surface modification steps, making large-scale preparation of stable microcapsules technically demanding. As a result, most studies remain at the laboratory or proof-of-concept stage.
3. Interfacial compatibility among components. The mismatch between the core–shell structure and photo-responsive fillers can lead to leakage or degradation during thermal cycling. Improving interfacial bonding via surface modification, coupling agents, or gradient-interface engineering will enhance durability.
4. Limited multifunctional integration. Current studies primarily focus on the development of single- or dual-function MEPCMs. Future research should aim to achieve three or more functional properties by incorporating multiple types of functional materials.
5. Scalability and practical implementation. The industrial deployment of photo-responsive MEPCMs still faces challenges in terms of large-scale synthesis, process costs, and long-term reliability. Simplifying fabrication routes, improving encapsulation efficiency, and adopting environmentally sustainable methods will be essential to enable scalable production and real-world integration.

Looking forward, among the photo-responsive MEPCMs systems discussed, carbon-based photothermal MEPCMs currently exhibit the most promising potential for near-term industrial applications due to their mature synthesis routes, superior photothermal conversion efficiency, high stability, and relatively low cost. In contrast, photocatalytic MEPCMs are still largely limited to ultraviolet-light responsiveness, and their overall energy conversion efficiency remains modest. Emerging photocatalytic materials, such as perovskites and covalent organic frameworks (COFs), show excellent light absorption and charge transfer characteristics. However, their integration with MEPCMs has been rarely explored, and systematic investigations on such composites remain scarce. Photoluminescent MEPCMs, on the other hand, are still in their early exploratory stage, mainly constrained by high material costs and relatively narrow application domains.

Future progress toward commercialization and large-scale implementation of photo-responsive MEPCMs will rely on addressing several critical challenges, including process scalability, long-term interfacial stability, and cycling durability of both materials and structures. Building sustainable and reproducible fabrication frameworks is essential to bridge the gap between laboratory research and practical deployment of these advanced MEPCMs in energy storage, smart coatings, and building materials.

## Figures and Tables

**Table 1:** Comparison between organic and inorganic PCMs under different operating conditions.

Category	Typical Materials	Melting Range	Advantages	Limitations	Representative Applications
Organic PCMs	Paraffins [34,35], polyols, fatty acids [36,37]	Low- to medium-temperature (typically <200 °C)	Chemically stable, non-corrosive, low cost, adjustable melting point	Low thermal conductivity, flammable, low density	Building thermal regulation, textiles, packaging
Inorganic PCMs	Salt hydrates [40], metals and their alloys [41,42,43,44]	Low- to high-temperature (approximately 10–800 °C)	High latent heat per volume, high thermal conductivity, non-flammable	Corrosive, prone to supercooling or phase separation, high cost	Industrial heat recovery, solar thermal systems, electronics cooling

## Data Availability

No new data were created or analyzed in this study. Data sharing is not applicable to this article.

## Abbreviations

The following abbreviations are used in this manuscript:

ACQ	Aggregation-caused quenching
BS	Butyl stearate
CA	Capric acid
CB	Carbon black
CDQs	Carbon quantum dots
CNC	Cellulose nanocrystal
CNT	Carbon nanotube
CS	Chitosan
CTAB	Cetyl trimethyl ammonium bromide
DPP	Diketopyrrolopyrrole
DSC	Differential scanning calorimetry
EC	Ethyl cellulose
GO	Graphene oxide
HF	Hydrofluoric acid
HLB	Hydrophilic and lipophilic balance
ICG	Indocyanine green
LA	Lauric acid
LHS	Latent heat storage
LSPR	Localized surface plasmon resonance
LTH	Light-to-hydrogen
MB	Methylene blue
MEPCMs	Microencapsulated phase-change materials
MF	Melamine-formaldehyde
MIP	Molecularly imprinted polymer
NG	Nano-graphite
PA	Paraffin
PANI	Polyaniline
PB	Prussian blue
PCMs	Phase-change materials
PDA	Polydopamine
PMMA	Polymethyl methacrylate
PPY	Polypyrrole
PS	Polystyrene
PU	Polyurethane
PUA	Polyurethane acrylates
PVP	Polyvinylpyrrolidone
RhB	Rhodamine B
rGO	Reduced graphene oxide
SDB	Styrene divinylbenzene
SDBS	Sodium dodecyl-benzenesulfonate
SHS	Sensible heat storage
TCHS	Thermal chemical heat storage
TEOS	Tetraethoxysilane
TES	Thermal energy storage
UF	Urea-formaldehyde
Vis-NIR	Visible-to-near-infrared
